# MicroRNA-141 inhibits tumor growth and metastasis in gastric cancer by directly targeting transcriptional co-activator with PDZ-binding motif, TAZ

**DOI:** 10.1038/cddis.2014.573

**Published:** 2015-01-29

**Authors:** Q-F Zuo, R Zhang, B-S Li, Y-L Zhao, Y Zhuang, T Yu, L Gong, S Li, B Xiao, Q-M Zou

**Affiliations:** 1 National Engineering Research Center of Immunological Products, Department of Microbiology and Biochemical Pharmacy, College of Pharmacy, Third Military Medical University, Chongqing 400038, PR China; 2 Research and Service Center of Laboratory Medicine, ChengDu Military General Hospital, Chengdu 610083, PR China; 3 General Surgery and Center of Minimally Invasive Gastrointestinal Surgery, Southwest Hospital, Third Military Medical University, Chongqing 400038, PR China

## Abstract

Gastric cancer (GC) is a biologically heterogeneous disease accompanying various genetic and epigenetic alterations, and the molecular mechanisms underlying this disease are complex and not completely understood. Increasing evidence shows that abnormal microRNA (miRNA) expression is involved in GC tumorigenesis, but the role of specific miRNAs involved in this disease remains elusive. MiR-141 was previously reported to act as tumor suppressors or oncogenes in diverse cancers. However, their accurate expression, function and mechanism in GC are largely unclear. Here we found that the expression of miR-141 was significantly reduced in GC compared with paired adjacent normal tissues and was significantly correlated with a more aggressive phenotype of GC in patients. Ectopic expression of miR-141 mimics in GC cell lines resulted in reduced proliferation, invasion and migration, and inhibition of miR-141 in GC cell lines promoted cell proliferation, invasion and migration *in vitro*. We further demonstrated that miR-141 acted as tumor suppressors through targeting transcriptional co-activator with PDZ-binding motif (TAZ) in GC. Moreover, the inverse relationship between miR-141 and its target was verified in patients and xenograft mice. Finally, overexpression of miR-141 suppressed tumor growth and pulmonary metastasis in nude mice. Take together, we identified that miR-141 is a potent tumor suppressor in the stomach, and its growth inhibitory effects are, in part, mediated through its downstream target gene, TAZ. These findings implied that miR-141 might be employed as novel prognostic markers and therapeutic targets of GC.

Gastric cancer (GC) is the fourth most common cancer and the second leading cause of cancer-related death in the world. It is estimated that 4 64 000 men and 2 73 000 women will have died from GC in 2011.^1, 2^ The pathogenesis of GC is complex and related to multiple factors. *Helicobacter pylori* infection is a WHO class I carcinogen for the development of antral/body GC and diet high in salt and nitrate are synergistic risk factors for GCs.^3^ Interactions among host, environmental and bacterial factors influence the disease outcome.^4, 5^ According to the National Cancer Institute, the incidence of GC is highest in East Asia. In China, the incidence of GC is still high despite advances in treatment and subsequent improvement in prognosis.^6^ Clinically, the absence of specific symptoms renders early diagnosis of this deadly disease difficult. Gastrectomy remains the mainstay treatment for GC, but the prognosis for advanced stage patients is still very poor.^7^ Thus an improved and detailed understanding of the mechanisms underlying GC development and progression is urgently needed.

Recent discoveries have shed new light on the involvement of a class of non-coding RNA known as microRNA (miRNA) in GC. MiRNAs are a group of non-coding, small (approximately 22 nt in length) RNAs that have important roles in the pathogenesis of human diseases by modulating the activity of specific mRNA targets.^8^ This large family of highly conserved small non-coding RNAs may regulate a vast number of protein-coding genes, including oncogenes and tumor-suppressor genes, which suggest that miRNAs can function as tumor suppressors or oncogenes.^9, 10^ There are an increasing number of studies showing the overexpression or downregulation of specific miRNA in GC.^11^ MicroRNA-21 promotes tumor proliferation and invasion in GC by targeting phosphatase and tensin homolog and is a new marker of circulating tumor cells in GC patients.^12^ MiRNA-223 promotes GC invasion and metastasis by targeting tumor-suppressor erythrocyte membrane protein band 4.1-like 3.^13^ MiR-29c acts as a tumor suppressor in GC by directly targeting integrin beta-1. Loss of miR-29c expression is an early event in the initiation of gastric carcinogenesis.^14^ Our previous studies showed that miR-25 was overexpressed in plasma and primary tumor tissues of GC patients and that miR-25 promotes GC migration, invasion and proliferation by directly targeting transducer of ERBB2 and ERBB1.^15^ These studies suggested a close correlation between miRNAs and the development, progression, metastasis and prognosis of GC. MiR-141 is located from 6964097 bp to 6964191 bp on chromosome 12p13 and belongs to the microRNA-200 family. Extensive research has shown that miR-141 deregulated as a tumor suppressor in many tumors, including ovarian cancer,^16, 17^ hepatocellular carcinoma,^18^ renal cell carcinoma,^19^ colorectal cancer and pancreatic cancer.^20, 21^ Despite an increasing number of studies on the biogenesis and mechanisms of miR-141 in the pathogenesis of GC,^22, 23, 24^ the accurate expression and mechanistic function of them in GC are largely unclear.

Transcriptional coactivator with PDZ-binding motif (TAZ), also known by gene name WW domain-containing transcription regulator 1 (WWTR1),^25^ is a transcription cofactor that has pivotal roles in epithelial-to-mesenchymal transition (EMT), cell growth and organ development.^26^ TAZ functions by transactivating numerous transcription factors, including RUNX, PPARγ, Pax3, TBX5 and TTF-1.^27, 28, 29, 30^ The regulation of TAZ and its paralog, Yes-associated protein (YAP), occurs primarily via Hippo signaling tumor-suppressor pathway.^26^ Recent studies showed that TAZ is a regulator of CSC-like properties in breast cancer and mesenchymal transition in malignant glioma.^31, 32^

Here we monitored miRNAs and mRNA expression profiles of GC and identified miR-141 as one of the most significantly downregulated miRNAs in GC tissues and a critical suppressor of GC cell proliferation and metastasis both in *in vitro* and *in vivo* studies. We also showed that miR-141 may function as a tumor suppressor by directly targeting TAZ.

## Results

### MiR-141 is downregulated in primary tumor tissues of GC and correlates inversely with metastatic capacity in GC tissue

**Figure 1:**
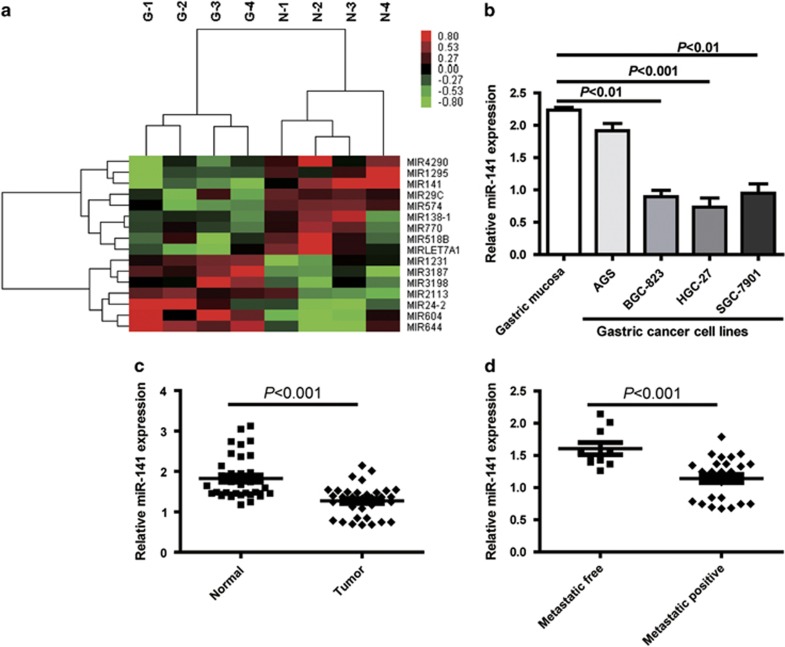
MiR-141 is downregulated in primary tumor tissues of GC and correlates inversely with metastatic capacity in GC tissue. (**a**) Differential expression of 16 miRNAs between paired GC and NM tissues in microarray analysis. (**b**) MiR-141 expression in gastric mucosa tissues of three healthy subjects and GC cell lines of AGS, BGC-823, HGC-27 and SGC-7901. Data are presented as mean±S.D. (*n*=3). (**c**) Expression status of miR-141 in an independent validation cohort of 36 pairs of matching GC and NM tissues (paired *t*-test). (**d**) qRT-PCR data of miR-141 levels in primary GC (metastasis positive or metastasis free) lymph node metastasis. RNU6B serve as an internal reference. All assays were performed in duplicates

To discover the expression of miRNAs transcriptomes that distinguish GC from non-cancerous gastric tissues, microarray analysis of small RNAs was performed on four pairs of frozen GCs and adjacent normal tissues. Using paired *t*-test, 16 miRNAs were found to be differentially expressed between GC and normal mucosa (NM) tissues (*P*≤0.05 with ≥1.5 fold change; Figure 1a). In order to further confirm the robustness of our GC miRNA signature discovered by microarray analysis, we compared our miRNA expression results with other independent miRNA expression profiles that were generated from GCs.^33^ By comparing our miRNA expression results with the independent miRNA profiles, we identified four miRNAs (miR-141, miR-29c, miR-24 and let-7a) that were shared between these data sets and were differentially expressed in GCs. Gomes *et al.*^34^ reported that miR-141 was one of the specific expression signature among nine miRNAs, which clustered the samples into two groups: healthy tissue and GC tissue. As miR-141 was one of the most significantly downregulated miRNA in GCs *versus* NMs and was shared between our miRNA profiles and other independent profiles, we selected this miRNA for further validation in GC cell lines and gastric tissues. In terms of validation, we quantified the expression of miR-141 in 36 pairs of frozen GCs and matched NMs, as well as in four GC cell lines. The quantitative real-time PCR (qRT-PCR) analyses showed that miR-141 levels were specifically attenuated in GC cell lines (BGC-823, HGC-27 and SGC-7901), compared with normal gastric mucosa (Figure 1b), and revealed that miR-141 expression was significantly downregulated in GC tissues, compared with paired NM tissues (*P*<0.001; Figure 1c). To further investigate the clinicopathological and prognostic significance of miR-141 levels in patients with GC, the levels of miR-141 in 36 pairs of GC tissues were statistically analyzed. When the 36 tumors were stratified, based on clinical progression, miR-141 expression was found to be diminished in primary tumors that subsequently metastasized compared with those that did not metastasize (*P*<0.001; Figure 1d). However, no significant differences were observed in age, gender, tumor location or histology. These results suggest that miR-141 is consistently downregulated in GCs, and may serve as tumor suppressor in this disease, and miR-141 correlates inversely with metastatic capacity in GC tissues.

### MiR-141 expression inhibits cell proliferation, invasion and migration in GC cells

**Figure 2:**
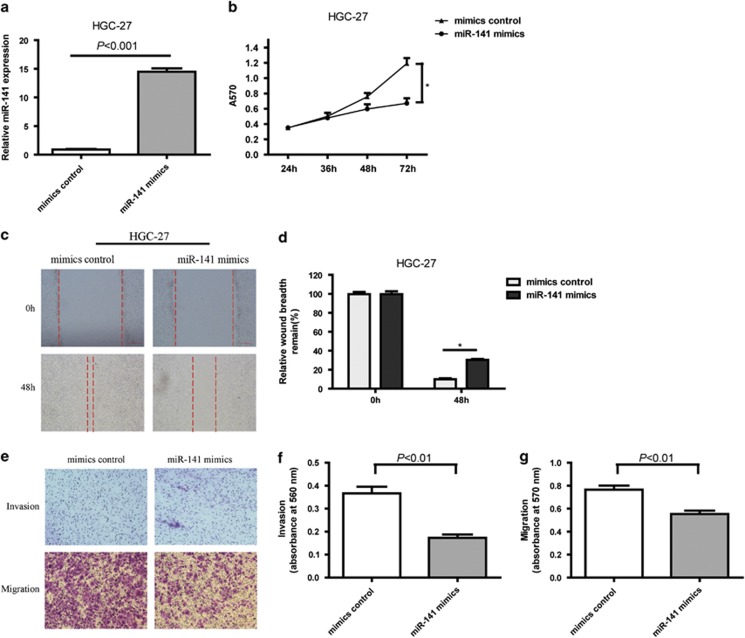
The effect of ectopic expression of miR-141 levels on cell proliferation, migration and invasion of HGC-27 *in vitro*. (**a**) MiR-141 effectively increased miR-141 expression level, as determined using real-time PCR in HGC-27 cell line, which was normalized against U6 RNA. Data are presented as mean±S.D. (*n*=3). (**b**) Overexpression of miR-141 by miR-141 mimics affected the cell proliferation of HGC-27 cells as determined using the CCK8 assay. Data are presented as mean±S.D. (*n*=6). (**c** and **d**) Overexpression of miR-141 by miR-141 mimics affects the migration capacity of HGC-27 cells as determined using the wound-healing assay. Representative images were captured at 0 and 48 h after transfection. The relative wound breadth remaining (100%) represents the migration capacity of GC cells, and the breadth at 0 h was set as 100%. All of the experiments were performed three times. Data are presented as mean±S.D. (*n*=3). *P<0.05. (**e**) Representative images and (**f** and **g**) bar graphs depicting the invasion and migration ability of HGC-27 after mimics control or miR-141 mimics 24 h after transfection. Data are presented as mean±S.D. (*n*=6)

To better understand the mechanistic role of miR-141 in gastric carcinogenesis, GC cell lines were transfected with either a miR-141 mimics or a mimics control. As BGC-823, HGC-27 and SGC-7901 cell lines expressed miR-141 at low levels, we selected HGC-27 for transfection experiments, as this cell line has been well characterized (Figure 1b). Overexpression of miR-141 was confirmed using real-time PCR as shown in Figure 2a. To determine the role of miR-141 in the proliferation of GC cells *in vitro*, Cell Counting Kit 8 (CCK8) assay were performed, as shown in Figure 2b. Overexpression of miR-141 inhibited cell proliferation in HGC-27 at 72 h after transfection. Then we analyzed the effect of ectopic miR-141 expression on cellular invasion and migration potential of HGC-27 cells. In the wound-healing assay, HGC-27 cells in the miR-141 mimics group migrated more slowly. High miR-141 expression significantly suppressed the ability of the cells to migrate (Figures 2c and d). In the transwell invasion and migration assay, cells transfected with miR-141 mimics displayed an inhibition in invasion and migration ability when compared with the control group in HGC-27 cells (Figures 2e–g). Collectively, the results indicated that ectopic miR-141 significantly reduced cell proliferation, migration and invasion *in vitro*.

### Inhibition of miR-141 promotes cell proliferation, invasion and migration in GC cells *in vitro*

**Figure 3:**
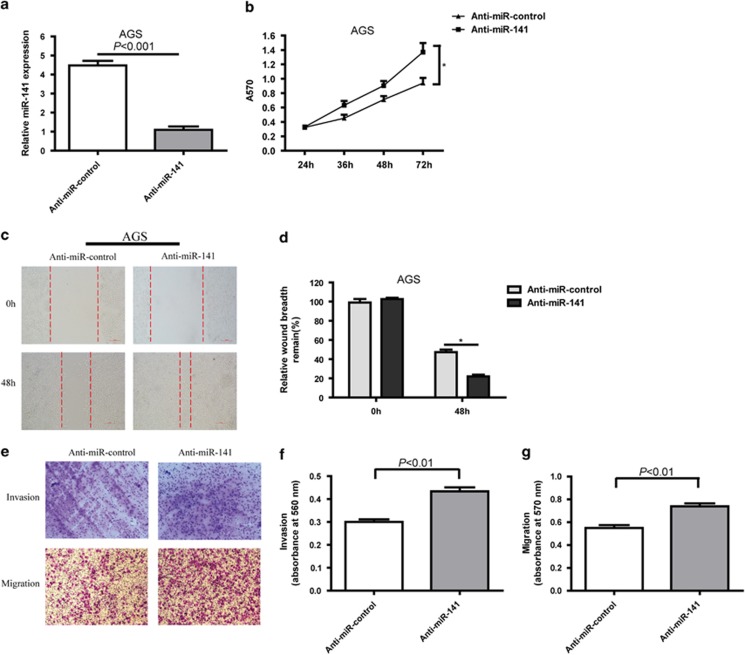
The effect of inhibition of miR-141 levels on cell proliferation, migration and invasion of AGS *in vitro*. (**a**) MiR-141 inhibitors effectively decreased miR-141 expression level, as determined using real-time PCR in AGS cell line, which was normalized against U6 RNA. Data are presented as mean±S.D. (*n*=3). (**b**) Inhibition of miR-141 by miR-141inhibitors affected the cell proliferation of AGS cells as determined using the CCK8 assay. Data are presented as mean±S.D. (*n*=6). (**c** and **d**) Inhibition of miR-141 by miR-141 inhibitors affects the migration capacity of AGS cells as determined using the wound-healing assay. Representative images were captured at 0 and 48 h after transfection. The relative wound breadth remaining (100%) represents the migration capacity of GC cells, and the breadth at 0 h was set as 100%. All of the experiments were performed three times. Data are presented as mean±S.D. (*n*=3). *P<0.05. (**e**) Representative images and (**f** and **g**) bar graphs depicting the invasion and migration ability of AGS after anti-miR-control or anti-miR-141 24 h after transfection. Data are presented as mean±S.D. (*n*=6)

It was then determined whether miR-141 inhibited cell proliferation, invasion and migration of AGS GC cell line, in which miR-141 expression was not significantly changed when compared with normal gastric mucosa tissues. MiR-141 was transiently inhibited in AGS cells with anti-miR-141 (Figure 3a). Then we analyzed the effect of inhibition of miR-141 levels on cellular proliferation, invasion and migration potential of AGS cells. The results showed that the suppression of miR-141 enhanced cell proliferation (Figure 3b), invasion and migration (Figures 3c–g).

### MiR-141 directly regulates TAZ in GC cells

**Figure 4:**
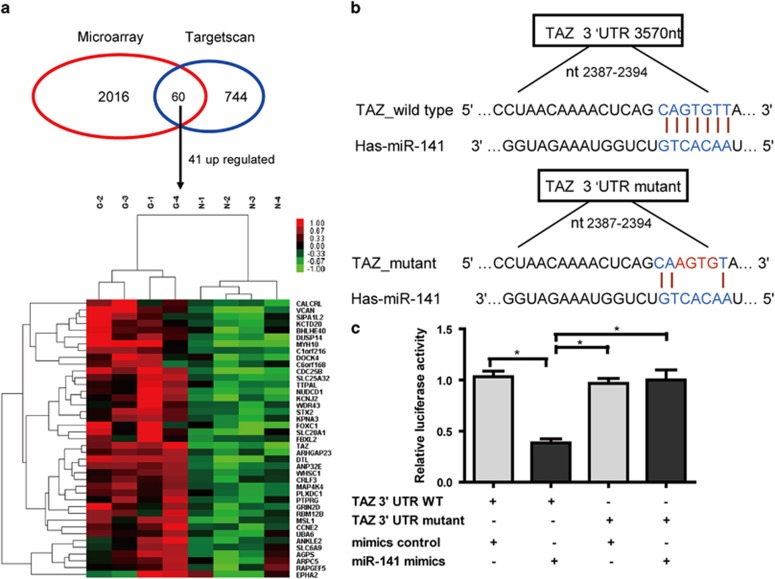

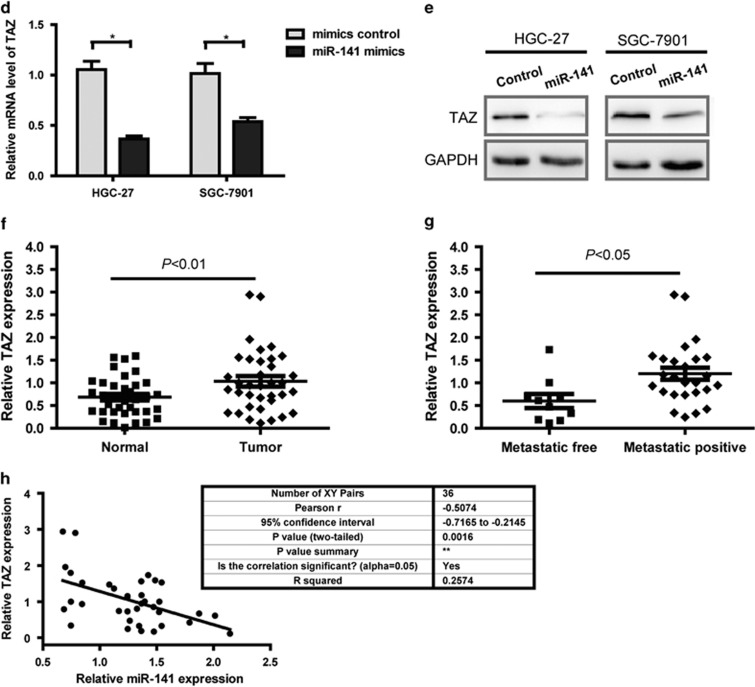
Continued

As the function of miRNAs in tumor development is dependent on targeting their key target genes, it is crucially important to identify the targets of miR-141. A reverse correlation in expression profiles between a miRNA and corresponding predicted targets increases the confidence of the conditional miRNA–target interaction. Based on these observations, we combined differentially expressed miRNAs and target genes, downregulated (upregulated) miRNAs *versus* upregulated (downregulated) targets, to identify the miRNA-regulated target genes in GC. Candidate targets were first determined using target prediction engine TargetScan. Then we examined the mRNA expression profiles in four pairs of primary tumor tissues of GC patients with and matched non-tumor tissues by microarray analysis. The results showed that 2016 mRNAs were differently regulated (fold change ≥1.5 and a *P*-value ≤0.05) (data not shown). When compared with the predicted targets by TargetScan, 41 target genes that have miR-141 seed sites were predicted via miRNA target prediction tools (TargetScan) and microarray analysis (upregulated expression of protein-coding genes between paired GC and NM tissues) (Figure 4a). Among these candidate targets, TAZ was predicted as a novel target of miR-141 and was selected as our target gene in GC for further study, as it seemed more germane to present study and has been shown to associate with prognosis and metastasis in patients with breast cancers. To determine whether TAZ are direct targets of miR-141, wild-type and mutant 3′-untranslated region (3′-UTR) lacking miR-141 binding sites were cloned into the downstream of firefly luciferase coding region in pGL-3 luciferase reporter vector. The constructs were then cotransfected with pRL-TK and miR-141 mimics or mimics control into HEK293T cells, respectively. The relative luciferase activity was reduced by 55% in pGL-3 vectors with wild-type TAZ 3′-UTR but not in those with respective mutant 3′-UTRs (Figures 4b and c). To further determine whether miR-141 can decrease endogenous TAZ expression, we transfected miR-141 mimics in HGC-27 and SGC-7901 cells. As shown in Figures 4d and e, Overexpression of miR-141 by transfecting miR-141 mimics resulted in a significant reduction in TAZ mRNA transcription as well as protein expression by western blotting. To confirm the relationship between miR-141 and TAZ expression, miR-141 and TAZ mRNA expression levels were investigated in the 36 primary GC and paired adjacent normal tissues. The results showed that the mRNA levels of TAZ were significantly elevated in GC tissues compared with paired adjacent normal tissues (Figure 4f), and the expression of TAZ in patients with lymphatic metastasis were 2.01 times higher than those in patient without lymphatic metastasis, and the differences were statistically significant (*P*<0.05) (Figure 4g). Moreover, the mRNA levels of TAZ were negatively correlated with miR-141 levels in the 36 primary GC tissues (Figure 4h).

### *In vitro* functional analysis and expression of TAZ in GC cells and the ectopic expression of TAZ restores the effects of miR-141 on cell proliferation, migration and invasion in GC cells

**Figure 5:**
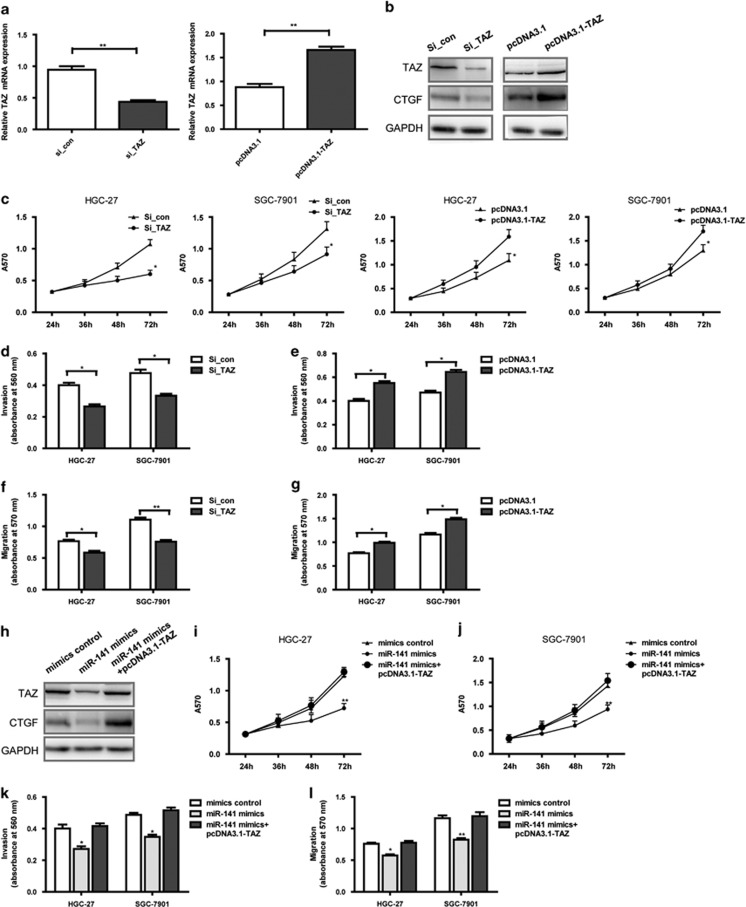
*In vitro* functional analysis and expression of TAZ in GC cells, and ectopic expression of TAZ restores the effects of miR-141 on cell proliferation, migration and invasion in GC cells. (**a**) qRT-PCR assays show the mRNA expression of TAZ in GC cells transfected with si_con, si_TAZ, pcDNA3.1 vector or pcDNA3.1-TAZ vector, respectively. Data are presented as mean±S.D. (*n*=3). **P<0.01. (**b**) Western blotting assays show the protein expression of TAZ and CTGF in GC cells transfected with si_con, si_TAZ, pcDNA3.1 vector or pcDNA3.1-TAZ vector, respectively. GAPDH served as an internal reference. (**c**–**g**) Cell proliferation ability, invasion ability and migration ability assays after HGC-27 and SGC-7901 cells were transfected with si_con, si_TAZ, pcDNA3.1 vector or pcDNA3.1-TAZ vector, respectively. Histogram revealed the values of absorbance at 570 nm for migration or at 560 nm for invasion. The assays were repeated in duplicates. Data are presented as mean±S.D. (*n*=6). *P<0.05. (**h**) Western blotting assays show the protein expression of TAZ and CTGF in GC cells after transfection with mimics control, miR-141 mimics or cotransfection with miR-141 mimics and pcDNA3.1-TAZ vector. GAPDH served as an internal reference. (**i**–**l**) pcDNA3.1-TAZ counteracts the inhibition of growth, migration and invasion of HGC-27 and SGC-7901 cells caused by miR-141 compared with that observed in control cells. Histogram revealed the values of absorbance at 570 nm for migration or at 560 nm for invasion. Data are presented as mean±S.D. (*n*=6). *P<0.05. The assays were repeated in duplicates

TAZ has been identified as a component of an emerging Hippo signaling pathway that has important roles in regulating cell proliferation, apoptosis, tumor formation and organ size in both Drosophila and mammals. To determine whether miR-141 inhibited tumor phenotypes of GC in a TAZ-dependent manner, the effects of small interfering RNA (siRNA) targeting of TAZ and overexpression of TAZ protein with pcDNA3.1-TAZ were examined. The results showed that suppression of TAZ expression by siRNA reduced TAZ mRNA and protein levels and TAZ target CTGF (connective tissue growth factor) protein levels and inhibited cell proliferation, invasion and migration (Figures 5a–g), which is consistent with the inhibitory effects induced by miR-141. To avoid misinterpretations of the off-target effects as true effects of TAZ knockdown by siRNA, we have used an additional interfering RNA (siRNA#2) to suppress the expression of TAZ. As expected, the results showed that suppression of TAZ expression by siRNA#2 reduced TAZ mRNA and protein levels and TAZ target CTGF protein levels (Supplementary Figures 1A and B) and inhibited cell proliferation, invasion and migration (Supplementary Figures 1C–F). CTGF regulates cell adhesion, proliferation and migration, as a direct target of TAZ and TEAD.^35^ The ectopic expression results showed that overexpression of TAZ protein with pcDNA3.1-TAZ enhanced TAZ mRNA and protein levels and TAZ target CTGF protein levels and promoted cell proliferation, invasion and migration (Figures 5a–g), and, as predicted, TAZ and TAZ target CTGF expression were markedly decreased in the GC cells after transfection with miR-141 and were restored when the GC cells were cotransfected with pcDNA3.1-TAZ and miR-141 mimics (Figure 5h). Function investigation showed that the co-transfection of pcDNA3.1-TAZ and miR-141 mimics into HGC-27 and SGC-7901 cells significantly rescued miR-141-suppressed proliferation, migration and invasion (Figures 5i–l). These findings demonstrated that miR-141 impairs the proliferation, migration and invasion of GC cells via the miR-141/TAZ signaling axis.

### MiR-141 inhibited the growth of HGC-27-engrafted tumors and repressed the distal pulmonary metastases *in vivo*

**Figure 6:**
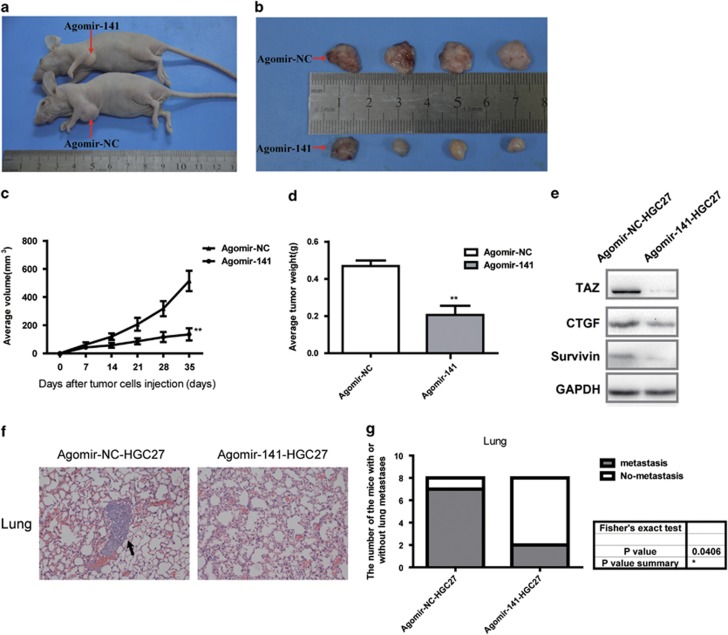
Overexpression of miR-141 inhibits GC growth and represses the distal pulmonary metastases *in vivo*. (**a** and **b**) Photographs of mice injected with agomir-NC-HGC27 or agomir-141-HGC27. (**c**) Graph representing tumor volumes at the indicated days during the experiment for the two groups: agomir-NC and agomir-141, six mice in each group. Data are presented as mean±S.D. (*n*=6). **P<0.01. (**d**) Tumor weight averages between agomir-NC-HGC27 and agomir-141-HGC27 mice groups at the end of the experiment (day 35). Data are presented as mean±S.D. (*n*=6). **P<0.01. (**e**) Immunoblot analysis of TAZ, CTGF and survivin expression in tumors from xenograft mice. (**f**) HE staining of lung tissue isolated from nude mice that had been injected with either agomir-NC-HGC27 or agomir-141-HGC27 via lateral tail veins ( × 200 magnification). The metastasis nodules are indicated by arrows. (**g**) The graph gives the incidences of metastasis in mice that had received lateral tail injections of each cell line. Eight mice in each group, Fisher's exact test: *P<0.05

To further investigate the contribution of miR-141 *in vivo*, we selected HGC-27 cell which possesses the lowest expression of miR-141 to perform the tumor xenograft studies and pulmonary metastasis via BALB/c nude mice models. The HGC-27 cells were collected after transfection with high dose of agomir-NC (5 *μ*M) or agomir-141 (5 *μ*M) for 24 h. For tumor xenograft studies, HGC-27 cells transfected with agomir-NC (5 *μ*M) or agomir-141 (5 *μ*M) were injected subcutaneously into the axillary fossae of the female nude mice. After 5 weeks, the volumes of the tumors resulting from injection were significantly smaller than those resulting from agomir-NC-HGC27 (Figures 6a–c). In agreement with the tumor volumes, the weights of tumors from the agomir-141-HGC27 group were significantly lower than the agomir-NC-HGC27 group (Figure 6d). In addition, immunoblot analyses revealed that tumors from agomir-141-HGC27 cells had reduced TAZ, TAZ target CTGF and survivin protein levels compared with agomir-NC-HGC27 cells (Figure 6e). CTGF and survivin are known as TAZ targets, which facilitate cancer progression through promoting proliferation.^36^ For pulmonary metastasis assays, the HGC-27 cells transfected with agomir-NC or agomir-141 were injected into nude mice through the lateral tail vein, respectively. After 5 weeks, examination of the lungs clearly revealed that the number of mice with lung metastases was lower in the group injected with agomir-141-HGC27 cells compared with the group injected with the agomir-NC-HGC27 cells (Figures 6f and g). Together, the data suggest that miR-141 inhibit the growth of HGC-27-engrafted tumors and repress the distal pulmonary metastases *in vivo*.

## Discussion

In the present study, microarray analysis and qRT-PCR validation results showed that miR-141 expression was significantly decreased in GC tissues compared with the paired adjacent normal tissues. In addition, the downregulation of miR-141 was greater in lymph node metastases than in primary GC tissues. Furthermore, our results indicated that miR-141 directly targeted TAZ, leading to repressed cell proliferation and inhibited cell invasion and migration in GC cells. Moreover, our investigation for the expression of TAZ and miR-141 in 36 GC patients indicated that there was an inverse correlation between miR-141 and TAZ levels. Importantly, overexpressing miR-141 ameliorated progression of GC in an established experimental xenograft model and the distal pulmonary metastases. Using a series of *in vitro* and *in vivo* assays, we uncovered that miR-141 act as an important tumor suppressor in the normal gastric mucosa.

Previous studies have suggested that miR-141 has an important role in the regulation of human cancers. MiR-141 was identified to be downregulated in many cancers, including ovarian cancer,^16, 17^ hepatocellular carcinoma,^18^ renal cell carcinoma,^19^ colorectal cancer and pancreatic cancer.^20, 21^ MiR-141 is one member of the miR-200 family, a family that has been associated with the formation of cancer stem cells and regulation of the EMT.^20, 37, 38^ Liu *et al.*^18^ showed that miR-141 functioned as a tumor suppressor and inhibits the migration and invasion of HCC cells by targeting Tiam1. In a renal cell carcinoma study, miR-141 served as a potential biomarker for discriminating ccRCC from normal tissues and a crucial suppressor of ccRCC cell proliferation and metastasis by modulating the EphA2/p-FAK/p-AKT/MMPs signaling cascade.^19^ Apart from miR-141 downregulated in a large variety of human tumors, it is upregulated in the other types of human tumors. For instance, Mei *et al.*^39^ showed that miR-141 expression was significantly upregulated in NSCLC tissues and that its overexpression accelerated NSCLC cell proliferation *in vitro* and tumor growth *in vivo*. MiR-141 exhibits differential regulation in several circumstances and different subcellular distributions, therefore miR-141 expression may be oppositely changed in the development of different tumors. Although decreased miR-141 expression has been reported previously in human cancers, our study provides a novel and comprehensive insight into the functional role of miR-141 as it relates to the pathogenesis of GC. Gomes *et al.*^34^ identified miRNA expression profiles in GC using self-organizing maps, and miR-141 was confirmed as a prominent candidate miRNA dysregulated in GC. Chen *et al.*^22^ also showed that, compared with the normal gastric tissues, low levels of miR-141 expression were observed in cancerous tissue. This consistent finding from these independent cohorts as well as from different analysis tools was, in part, the rationale for selection and systematic exploration of the role of miR-141 in gastric neoplasia.

Most importantly, our results established TAZ as a direct functional effector of miR-141 in GC. TAZ was initially identified through its ability to interact with 14-3-3 proteins and is also called WWTR1.^28^ Sequence analysis revealed that TAZ shares homology with Yes-associated protein (YAP).^40^ TAZ and YAP are well known for their regulation by the Hippo pathway.^41^ TAZ has been recently proposed to endow self-renewal capacity to cancer stem cells.^31^ TAZ has also been identified as a component of an emerging Hippo-LATS tumor suppressor pathway that has important roles in regulating cell proliferation, apoptosis, tumor formation and organ size.^26^ Notably, elevated TAZ expression was observed in >20% breast cancer samples.^42^ Overexpression of TAZ causes morphological changes characteristic of cell transformation and promotes cell migration and invasion in human immortalized mammary epithelial cells, whereas knockdown of TAZ in breast cancer cells inhibits tumor formation.^42^ Cordenonsi *et al.*^31^ also demonstrated that the Hippo transducer TAZ confered cancer stem cell-related traits on breast cancer cells and was required to sustain self-renewal and tumor-initiation capacities in breast CSCs. Another two studies have connected TAZ overexpression with the development of the NSCLC and PTC (papillary thyroid carcinoma).^43, 44^ Up to now, the knowledge of functional roles and regulatory mechanism of TAZ in GC is still missing.

Consistent with these evidences that TAZ has been implicated in human tumorigenesis,^45^ our study showed an increased level of TAZ in GC tissues compared with NM tissues; moreover, TAZ were enriched in the primary GC tissues that inversely correlated with miR-141 levels. It is probable that the upregulation of TAZ by suppression of miR-141 contributed to tumor progression in GC. TAZ regulation by miR-141 was also examined in GC cell lines by western blotting and the luciferase reporter assay. Intriguingly, our mechanistic and functional data permit us to better appreciate the functional role of TAZ in human cancers. Its expression positively regulated GC cell proliferation, invasion and migration. Our results also indicated that TAZ knockdown suppressed GC cell growth by inducing proliferation, migration and invasion, which phenocopied the effects of miR-141 overexpression *in vitro*, and the ectopic expression of TAZ restores the effects of miR-141 on cell proliferation, migration and invasion in GC cells. These data clearly demonstrated that TAZ contributes to cell proliferation, invasion and migration in GC and is a direct and functional target of miR-141.

In conclusion, we identified that miR-141 is a potent tumor suppressor in the stomach, and its growth inhibitory effects are, in part, mediated through its downstream target gene, TAZ. To the best of our knowledge, this is the first study to demonstrate that the miR-141/TAZ axis regulates the proliferation, migration and invasion of GC cells. These findings provide a better understanding of the pathogenesis and development of GC and may be an important implication for future therapy of the GC.

## Materials and Methods

### Clinical samples

Thirty-six fresh GC tissue samples from GC patients and their matched adjacent non-tumor gastric mucosal tissues (>5 cm laterally from the edge of tumor region) were immediately snap-frozen in liquid nitrogen and stored at −80 °C until total RNA was extracted. The samples had been clinically and histopathologically diagnosed at the Southwest Hospital of Third Military Medical University (Chongqing, China) between 2010 and 2012 (Table 1). Tumor and non-cancerous tissues were confirmed histologically by hematoxylin and eosin staining. All samples were collected from consenting individuals according to the protocols approved by the Ethics Review Board at Third Military Medical University.

### Microarray analysis

The total RNA from fresh frozen GC tissues samples and their matched adjacent non-tumor gastric mucosal tissues was isolated using the Trizol reagent (Invitrogen, Carlsbad, CA, USA). The transcription expression profiles of microRNA and mRNA were performed with the Affymetrix GeneChip Human Gene 2.0 ST Array (Affymetrix Technologies, Santa Clara, CA, USA). Briefly, 1 *μ*g total RNA was labeled and hybridized following the manufacturer's protocol. Scanning was performed on a GeneChip Scanner 3000 7G, signals were extracted and the subsequent data processing was performed using Affymetrix Transcriptom Analysis Console (Affymetrix Technologies). The threshold we used to screen differentially expressed microRNAs or protein-coding RNA with statistical significance is fold change ≥1.5 and a *P*-value ≤0.05.

### Cell culture

GC cell lines, including HGC-27, SGC-7901, AGS and BGC-823, were obtained from American Type Culture Collection (Manassas, VA, USA) and cultured with DMEM/HIGH GLUCOSE (HGC-27, BGC-823), F12 (AGS) or RPMI 1640 (SGC-7901) medium (HyClone, Logan, UT, USA) supplemented with 10% FBS at 37 °C in 5% CO_2_, respectively. HEK293T cells were obtained from American Type Culture Collection and cultured in Dulbecco's modified Eagle's medium with 10% FBS.

### RNA extraction and qRT-PCR

Total RNA was extracted from the cultured GC cells, and tissues were harvested using the Trizol reagent (Invitrogen) according to the manufacturer's instruction. To measure the level of miR-141, qRT-PCR was performed by using Taqman probes (Invitrogen) in the Bio-Rad CFX96 real-time PCR system (Bio-Rad, Hercules, CA, USA) according to the manufacturer's instruction. The data were normalized using the endogenous U6 snRNA. For TAZ mRNA detection, reverse transcription was performed using the PrimeScript RT Master Mix (Perfect Real Time, TaKaRa, Dalian, China). Quantitative PCR was performed using SYBR Premix ExTaq II (TliRNaseH Plus; TaKaRa) in Bio-Rad CFX96 real-time PCR system. Glyceraldehyde-3-phosphate dehydrogenase (Gapdh) mRNA was used for normalization. Forward and reverse primers for TAZ (104 bp) and gapdh (226 bp) were 5′-ATCCCAGCCAAATCTCGTGA-3′ and 5′-GCCCTGCGGGTGGGT-3′ and 5′-GAAGGTGAAGGTCGGAGTC-3′ and 5′-GAAGATGGTGATGGGATTTC-3′, respectively. The 2^−ΔΔCT^ method was used in the analysis of PCR data.

### Cell proliferation assay

To measure the effect of miR-141 mimics, anti-miR-141, si_TAZ, si_TAZ#2 or pcDNA3.1-TAZ on cellular proliferation rates, AGS, HGC-27 and SGC-7901 cells were seeded at a density of 10^4^ per well in 96-well plates, respectively. The HGC-27 and SGC-7901 cells were transfected with mimics-control, miR-141 mimics, si_con, si_TAZ or si_TAZ#2 or cotransfected with miR-141 mimics and pcDNA3.1-TAZ, respectively, and AGS cells were transfected with anti-miR-control or anti-miR-141. Proliferation rates were analyzed using CCK8 (Beyotime, Shanghai, China) at 24, 48, 72 and 96 h posttransfection, and quantification was done on a microtiter plate reader (Bio-Rad) according to the manufacturer's protocol.

### Cell invasion and migration assays

HGC-27 and SGC-7901 cells were grown to 50–70% confluence and transfected with mimics-control, miR-141 mimics, si_con, si_TAZ or si_TAZ#2 or cotransfected with miR-141 mimics and pcDNA3.1-TAZ, respectively, and AGS cells were transfected with anti-miR-control or anti-miR-141. Twenty-four hours posttransfection, cells were seeded onto a Matrigel-coated membrane matrix (Millipore, Darmstadt, Germany) present in the insert of a 24-well culture plate. The migration and invasion of the cells were analyzed using the QCM Laminin Migration Assay (ECM220 Merck Millipore, Merck KGaA, Darmstadt, Germany) and Cell Invasion Assay Kit (ECM550 Merck Millipore, Merck KGaA). Absorbance was measured at 570 nm for migration or at 560 nm for invasion, according to the manufacturer's protocols.

### Constructs, reagents and assays

For 3′-UTR of TAZ, Complementary 58-mer DNA oligonucleotides containing the putative miR-141 target site within the 3′UTR of human TAZ mRNA were synthesized with flanking *Spe*I and *Hind*III restriction enzyme digestion sites (sense, 5′-CTAGTATAAAAAATTAAAAAAACAAGGGACCTAACAAAACTCAGCAGTGTTACTGTAT-3′ antisense, 5′-AGCTTATACAGTAACACTGCTGAGTTTTGTTAGGTCCCTTGTTTTTTTAATTTTTTAT-3′). Another construct containing mutant seed region was also generated as a control (CAGTGTT to CAAGTG). The DNAs and pMIR-REPORT Luciferace vectors were used to build the luciferase report vectors. The mutant 3′-UTR of TAZ served as a control. HEK293T cells were seeded onto 24-well plates (1 × 10^5^ cells per well) the day before transfections. Cells were transfected with wild-type pMIR-TAZ-3′UTR or mutant pMIR-TAZ-3′UTR (50 ng per well) and pRL-TK Renilla luciferase reports (10 ng per well), and then the cells were transfected miR-141 mimics or mimics control (50 nM). Cell lysates were prepared with the Passive Lysis Buffer (Promega, Madison, WI, USA) 48 h after transfection, and the luciferase activities were measured by using the Dual Luciferase Reporter Assay (Promega). The firefly luciferase activity was normalized to the renilla luciferase activity. For TAZ vector, homo sapiens full open reading frame cDNA clone for TAZ was transcribed, and the product was amplified by using primers with flanking *Spe* I and *Hind* III restriction enzyme digestion sites, followed by the DNAs inserted into the pcDNA3.1 vector (Invitrogen).

### Oligonucleotides and transfection

MiR-141 mimics, mimics control, anti-miR-control and anti-miR-141 molecules were obtained from RIBOBIO (Guangzhou, China) and transfected with Lipofectamine 2000 (Invitrogen) in AGS, HGC-27 or SGC-7901 cells at a final concentration of 50 nM. siRNAs (specifically for TAZ) and control siRNA were synthesized by RIBOBIO and transfected into HGC-27 or SGC-7901 cells (100 nM) using Lipofectamine 2000 (Invitrogen). Transfection of empty vectors pcDNA3.1 and pcDNA3.1-TAZ vectors was via Lipofectamine 2000 (Invitrogen). Transfection of agomir-NC and agomir-141 was via micrON miR-141 agomir kit (RIBOBIO) according to the manufacturer's protocol.

### *In vivo* tumor xenograft studies and metastasis assays

Female athymic BABL/c nude mice (6-weeks old) were used under conditions approved by the Institutional Animal Care and Use Committee of Third Military Medical University. To determine the proliferation capacity of miR-141 *in vivo*, 2 × 10^6^ HGC-27 cells transfected with agomir-NC (5*μ*M) or agomir-141 (5 *μ*M), respectively, were suspended in 200 *μ*l phosphate-buffered saline for each mouse and were injected subcutaneously into the axillary fossae of the female nude mice, six mice per group. Tumor diameters were measured every 7 days. At 35 days after injection, mice were killed, and tumors were weighted after necropsy. Tumor volume was calculated as follows: length × width^2^ × 1/2. For *in vivo* pulmonary metastasis assays, 1 × 10^6^ HGC-27 cells were transfected with agomir-NC (5 *μ*M) or agomir-141 (5 *μ*M), respectively. The cells were injected into the lateral tail veins of each anesthetized nude mice (eight per group, 6-week-old BALB/c-nu/nu). Five weeks after injection, the animals were killed; lungs were fixed with phosphate-buffered neutral formalin before paraffin embedding; and 5-*μ*m sections were stained with hematoxylin and eosin. The metastases were counted in a double-blind manner with the aid of a dissecting microscope (Nikon, Tokyo, Japan).

### Western blotting assay

Cultured cells were lysed in RIPA buffer, and the lysates were analyzed using the standard western blotting analyses. GAPDH that served as an internal reference were detected with anti-GAPDH rabbit monoclonal Abs (Cell Signaling Technology, Shanghai, China). The anti-TAZ, anti-CTGF, anti-survivin antibodies and horseradish peroxidase (HRP)-conjugated secondary antibody were obtained from Abcam (Hong Kong, China). Bound proteins were visualized by using the SuperSignal West Dura Extended Duration Substrate Kit (Thermo Scientific, Beijing, China).

### Statistical analysis

All data were analyzed by using the SPSS 17.0 software (SPSS Software lnc., Chicago, IL, USA), and graphs were generated using GraphPad Prism 5.0 (Graphpad Software Inc., La Jolla, CA, USA). Two-tailed Student's *t*-test was used to determine the differences between groups. The relationship between miR-141 and TAZ mRNA was analysed by correlation coefficients and linear regression analysis. Tumor lung metastases were analyzed by the Fisher's exact test. *P*≤0.05 was considered statistically significant.

## Figures and Tables

**Table 1:** Clinical and pathological characteristics of included patient samples

**Variable**	**Gastric cancer,** ***N*****=36**
*Gender*	
Male	20 (56)
Female	16 (44)
	
*Age, years*	
Median (range)	59 (32–74)
≥60	21 (58)
<60	15 (42)
	
*Tumor location*	
Body	17 (47)
Antrum	15 (42)
Cardia	4 (11)
Other	0
	
*Histology*	
Adenocarcinoma	28 (78)
Mucinous adenocarcinoma	8 (22)
Signet ring cell cancer	0
	
*TNM stage*	
I	6 (17)
II	8 (22)
III	16 (44)
IV	6 (17)
	
*Lymph node status*	
Metastasis	26 (72)
No metastasis	10 (28)

## Abbreviations

GC: gastric cancer
TAZ: transcriptional coactivator with PDZ-binding motif
WWTR1: WW domain-containing transcription regulator 1
CTGF: connective tissue growth factor
EMT: epithelial-to-mesenchymal transition
qRT-PCR: quantitative real-time PCR
3′-UTR: 3′-untranslated region
siRNA: small interfering RNA
GAPDH: glyceraldehyde-3-phosphate dehydrogenase
