# Supplementary material for: MicroRNA-141 inhibits tumor growth and metastasis in gastric cancer by directly targeting transcriptional co-activator with PDZ-binding motif, TAZ
Source: Cell Death Dis. 2015 Jan 29;6(1):e1623–. doi: 10.1038/cddis.2014.573 (PMC4669771; doi:10.1038/cddis.2014.573)
Supplement: Supplementary Information [file cddis2014573x1.doc]

Supplementary information for the manuscript:

# MicroRNA-141 inhibits tumor growth and metastasis in gastric cancer by directly targeting transcriptional co-activator with PDZ-binding motif, TAZ

# Q-F Zuo^1,4^, R Zhang^2,4^, B-S Li^1^, Y-L Zhao^3^, Y Zhuang^1^, T Yu^1^, L Gong^1^, S Li^1^, B Xiao^1^ and Q-M Zou^1^

^1^National Engineering Research Center of Immunological Products, Department of Microbiology and Biochemical Pharmacy, College of Pharmacy, Third Military Medical University, Chongqing 400038, PR China. ^2^ChengDu Military General Hospital, Chengdu 610083, PR China. ^3^General Surgery and Center of Minimally Invasive Gastrointestinal Surgery, Southwest Hospital, Third Military Medical University, Chongqing 400038, PR China.

Correspondence: Associated Professor B Xiao or Professor Q-M Zou

Department of Microbiology and Biochemical Pharmacy, College of Pharmacy, Third Military Medical University, NO.30, Gao Tan Yan Road, Sha Ping Ba District, Chongqing 400038, China.

E-mail: Binxiaotmmu@163.com or [Qmzou2007@163.com](mailto:Qmzou2007@163.com)

^4^These authors contributed equally to this work.

**Supplementary Figure Legends**

**Supplementary Figure 1. Downregulation of TAZ by transfection with si-TAZ#2 suppressed GC cell proliferation, invasion and migration.** (**A**) qRT-PCR assays show the mRNA expression of TAZ in GC cells transfected with si_con or si_TAZ#2. Data are presented as mean±S.D. (n=3). ***P* < 0.01. (**B**) Western blot assays show the protein expression of TAZ and CTGF in GC cells transfected with si_con or si_TAZ#2. GAPDH served as an internal control. (**C-F**) Cell proliferation ability, invasion ability and migration ability assays after HGC-27 and SGC-7901 cells were transfected with si_con or si_TAZ#2. Histogram revealed the values of absorbance at 570 nm for migration, or at 560 nm for invasion. The assays were repeated in duplicates. Data are presented as mean±S.D. (n= 6). **P* < 0.05, ***P* < 0.01.
