# Supplementary figures and images for: MicroRNA-141 inhibits tumor growth and metastasis in gastric cancer by directly targeting transcriptional co-activator with PDZ-binding motif, TAZ
Source: Cell Death Dis. 2015 Jan 29;6(1):e1623–. doi: 10.1038/cddis.2014.573 (PMC4669771; doi:10.1038/cddis.2014.573)

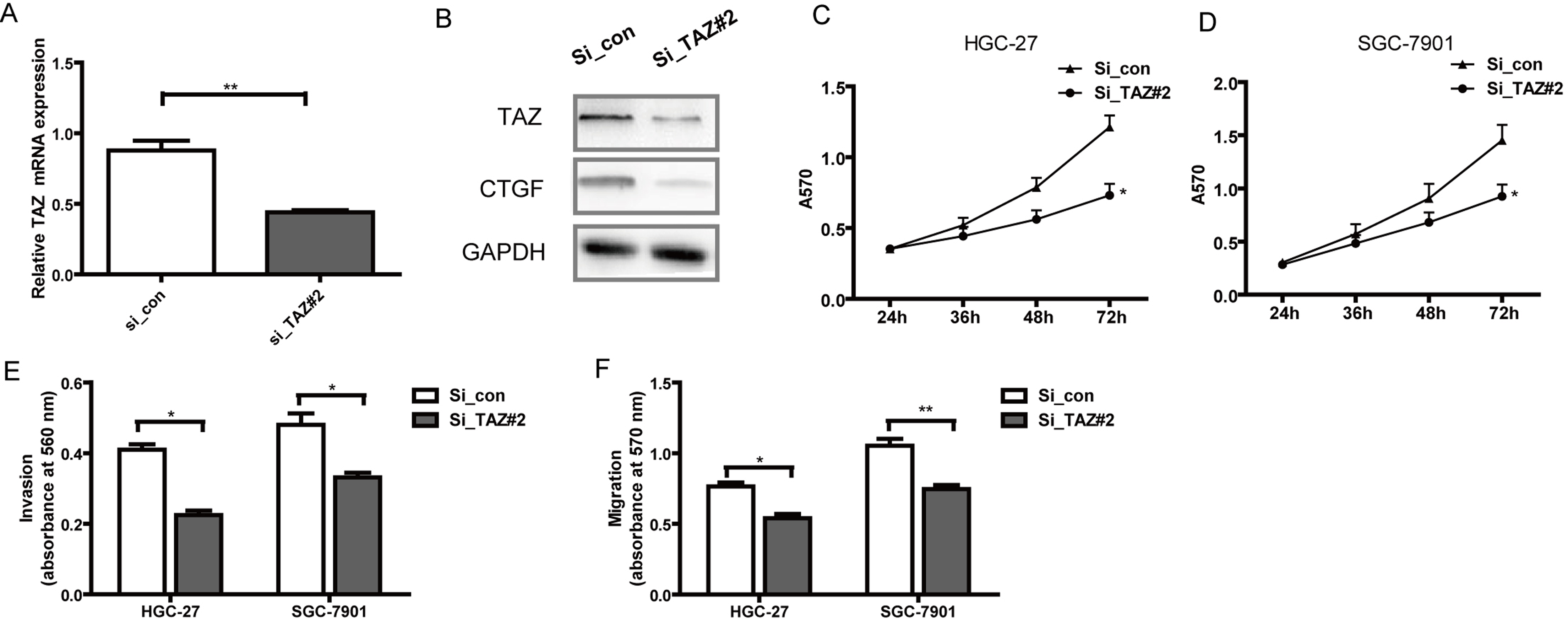

Supplement: Supplementary Figure 1 [file cddis2014573x2.tif]
